# The incidence and predictors of antibiotic-associated encephalopathy: a multicenter hospital-based study

**DOI:** 10.1038/s41598-024-59555-w

**Published:** 2024-04-16

**Authors:** Jean Hee Kim, Taewon Kim, Woojun Kim, Seong-Hoon Kim, Yun Jeong Hong, Eunyae Lim, Dae Woong Bae, Sang-Mi Noh, Jieun Lee

**Affiliations:** 1 Department of Neurology, Eunpyeong St. Mary’s Hospital, The Catholic University of Korea, Seoul, South Korea; 2 Department of Neurology, Incheon St. Mary’s Hospital, The Catholic University of Korea, #56 Dongsu-Ro, Bupyeong-Gu, Incheon, 21431 South Korea; 3 Department of Neurology, Seoul St. Mary’s Hospital, The Catholic University of Korea, Seoul, South Korea; 4 Department of Neurology, Uijeongbu St. Mary’s Hospital, The Catholic University of Korea, Seoul, South Korea; 5 Department of Neurology, Yeouido St. Mary’s Hospital, The Catholic University of Korea, Seoul, South Korea; 6 Department of Neurology, St. Vincent’s Hospital, The Catholic University of Korea, Seoul, South Korea; 7 Department of Neurology, Bucheon St. Mary’s Hospital, The Catholic University of Korea, Seoul, South Korea

**Keywords:** Microbiology, Neuroscience, Encephalopathy

## Abstract

This study aimed to evaluate the incidence and likelihood of antibiotic-associated encephalopathy (AAE), comparing rates among the classes of antibiotics in monotherapy or in combination therapy. We also investigated the associations between the incidence of AAE and the glomerular filtration rate (GFR) and electroencephalogram features. Consecutive admissions that used any kind of antibiotics to treat infectious diseases were identified from six hospitals. We classified antibiotics according to three distinct pathophysiologic mechanisms and clinical subtypes. We searched for the incidence of AAE as the primary outcome. A total of 97,433 admission cases among 56,038 patients was identified. Cases that received type 1 antibiotics had significantly more frequent AAE compared to those that received type 2 antibiotics (adjusted odds ratio [OR], 2.62; 95% confidence interval [CI] 1.15–5.95; *P* = 0.021). Combined use of type 1 + 2 antibiotics was associated with a significantly higher incidence of AAE compared to the use of type 2 antibiotics alone (adjusted OR, 3.44; 95% CI 1.49–7.93; *P* = 0.004). Groups with GFR < 60 mL/min/1.73 m^2^ had significantly higher incidence rates of AAE compared to those with GFRs ≥ 90 mL/min/1.73 m^2^ among cases that received type 1 + 2 antibiotics. Detection of spike-and-wave or sharp-and-wave patterns on electroencephalogram was significantly more common in the combination therapy group. Combination use of antibiotics was associated with a higher incidence of AAE compared to monotherapy. The incidence of AAE significantly increased as renal function decreased, and epileptiform discharges were more likely to be detected in cases receiving combined antibiotics.

## Introduction

Encephalopathy, a clinical syndrome referring to an alteration in mental status affecting cognition or level of arousal, can arise from diverse causes^1^. As a broad term encompassing a variety of clinical manifestations ranging from delirium to seizure, there are numerous conditions that contribute to encephalopathy, including primary neurologic conditions as well as systemic conditions such as exposure to toxins, drugs, metabolic disturbances, and infectious diseases^1^.

Infectious diseases, including pneumonia, influenza, and septicemia, are a major cause of death in those aged ≥ 65 years^2^, and antibiotics for treatment of infectious conditions are not only one of the most commonly prescribed medications in clinical practice, but also one of the most crucial treatments for combating life-threatening forms of these illnesses^3,4^. However, antibiotics are underrecognized as a cause of central nervous system (CNS) toxicity^5^. It is challenging to discern antibiotics from multiple potential causes of altered cognition, including fever, systemic inflammatory response, respiratory compromise, and renal/hepatic failure. However, identification of antibiotic-associated encephalopathy (AAE), especially when recognized and treated efficiently, is crucial in reversing the harm^6^.

Serious adverse CNS effects of antibiotics are reported with a frequency < 1%^7,8^. Recent reports on neurologic toxicity from antibiotics are mounting, with a rate of 15% associated with the use of the fourth-generation cephalosporin cefepime^5,6,9–12^. The incidence of cefepime-induced encephalopathy was higher especially in patients with lower values of glomerular filtration rate (GFR) and dialysis^13^. However, there are few large studies on the incidence of encephalopathy in clinical practice. Data describing the clinical features of and risk factors for AAE are limited to case reports and small series^7,14^.

A systematic review indicated that electroencephalograms (EEG) showed abnormalities in 70% of AAE cases and in nearly all cases of cephalosporin-associated encephalopathy where EEG data were available^9^. In this systematic review, the prevalence of epileptiform discharges or seizures on EEG in AAE varies by the classes of antibiotics. Notably, they were observed in 55% of cases of cephalosporin-associated encephalopathy, but in no cases of macrolide-, metronidazole-, or sulfonamide-associated encephalopathy^9^.

This study aimed to evaluate the incidence and likelihood of AAE in general clinical practice, especially in tertiary referral hospitals, with a comparison among the classes of antibiotics in monotherapy or in combination therapy. Second, we investigated the association between incidence of AAE and GFR. Finally, we explored EEG features according to the classes of antibiotics in patients with AAE.

## Results

### Baseline characteristics

**Figure 1:**
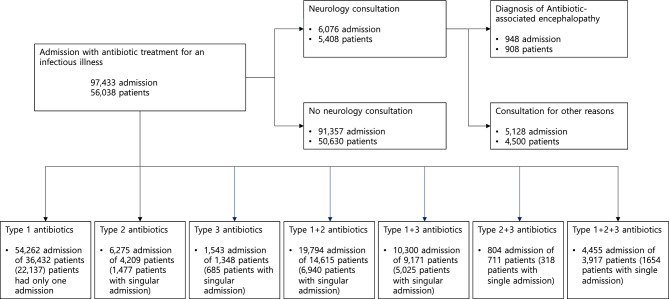
flow diagram for recruitment.

There were 97,433 admission cases among 56,038 patients identified to receive antibiotics during a consecutive 31 months (from June 1st, 2018, to December 31st, 2020) from the registered medical centers. Of the 97,433 admission cases, 41,395 (42.5%) cases were recurrent hospitalizations involving patients with multiple admissions during the study period. 38,236 distinct patients had only one hospitalization and were exclusively present in one group. The predominant type of antibiotics used was type 1 antibiotics followed by 1 + 2, type 1 + 3, type 2, type 1 + 2 + 3, type 3, and type 2 + 3 (Fig. 1).

There were 45,115 (46.3%) admission cases of female patients, and the mean age of all patients at admission was 60.0 ± 17.1 years. Demographics, clinical data, and initial laboratory results for each admission case are summarized in Table 1.

**Table 1:** Baseline characteristics of the total admission cases according to the subtypes of antibiotics.

	Type 1	Type 2	Type 3	Type 1 and 2	Type 1 and 3	Type 2 and 3	Type 1,2, and 3	Total	*p* value
Number of admission (N, %)	54,262 (55.7)	6275 (6.4)	1543 (1.6)	19,794 (20.3)	10,300 (10.6)	804 (0.8)	4455 (4.6)	97,433 (100)	
Number for unique patients (first admission) (N, %)	22,137 (58.9)	1477 (3.9)	685 (1.8)	6940 (18.2)	5025 (13.1)	318 (0.8)	1654 (4.3)	38,236 (100)	
Age (Mean, SD)	60.0 ± 16.5	56.8 ± 17.1	59.7 ± 18.1	61.5 ± 17.5	59.9 ± 18.6	55.7 ± 18.3	59.3 ± 18.6	60.0 ± 17.1	< 0.001
Female (N, %)	25,744 (47.4)	2975 (47.4)	702 (45.5)	8927 (45.1)	4517 (43.9)	395 (49.1)	1855 (41.6)	45,115 (46.3)	< 0.001
AAE (N, %)	457 (0.8)	20 (0.3)	12 (0.7)	298 (1.5)	83 (0.8)	1 (0.1)	77 (1.7)	948 (1.0)	< 0.001
WBC (X 10^9^/L)	8.9 ± 8.2 (N = 54,262)	7.9 ± 6.5 (N = 6275)	7.8 ± 4.0 (N = 1543)	9.8 ± 9.8 (N = 19,794)	9.9 ± 5.8 (N = 10,300)	8.0 ± 4.0 (N = 804)	10.2 ± 9.1 (N = 4455)	9.2 ± 8.1 (N = 97,433)	< 0.001
CRP (mg/dL)	13.5 ± 36.0 (N = 39,822)	13.1 ± 35.4 (N = 5306)	12.3 ± 28.8 (N = 1204)	23.5 ± 50.6 (N = 17,814)	24.2 ± 48.6 (N = 8065)	16.9 ± 36.3 (N = 696)	26.5 ± 53.1 (N = 4093)	17.7 ± 42.5 (N = 77,000)	< 0.001
ESR (mm/hr)	34.6 ± 29.7 (N = 25,494)	31.9 ± 30.0 (N = 4055)	35.7 ± 28.3 (N = 825)	42.3 ± 32.4 (N = 12,603)	38.9 ± 30.7 (N = 4475)	34.1 ± 28.6 (N = 454)	41.4 ± 32.8 (N = 3012)	37.1 ± 30.9 (N = 50,945)	< 0.001
†GFR (mL/min/1.73 m^2^)	84.7 ± 32.4 (N = 54,262)	88.2 ± 31.0 (N = 6275)	88.5 ± 29.1 (N = 1543)	83.8 ± 32.0 (N = 19,794)	88.5 ± 29.9 (N = 10,300)	86.4 ± 31.9 (N = 804)	86.9 ± 33.0 (N = 4455)	85.3 ± 32.0 (N = 97,433)	< 0.001

*AAE* Antibiotic-associated encephalopathy, *CRP* C-reactive protein, *ESR* erythrocyte sedimentation rate, *GFR* glomerular filtration rate, *WBC* white blood cell.

^†^GFR: calculated using 2021 CKD-EPI (chronic kidney disease-Epidemiology Collaboration) Creatinine. Values are mean ± standard deviation, and the number of patients (%). Chi-square test or Fisher’s exact test were used for categorical variables and analysis of variance (ANOVA) for continuous variables. (*p < 0.05, **p < 0.01).

In laboratory tests, the mean C-reactive protein (CRP) level (17.7 ± 42.5 mg/dL) and erythrocyte sedimentation rate (ESR) (37.1 ± 30.9 mm/h) at admission were above the upper normal reference limits of 0.5 mg/dL and 15 mm/h, respectively, and they were significantly higher in the combination therapy groups than in the monotherapy groups (P < 0.001).

### Incidence of AAE according to antibiotics used

There was significantly more frequent AAE in cases receiving type 1 + 2 antibiotics (298 cases, 1.5%) and type 1–3 antibiotics (77 cases, 1.7%) than those receiving other antibiotics (*P* < 0.001). There were 948 cases (1.0%) of AAE, including 457 (0.8%) cases affiliated with type 1 monotherapy, 20 cases (0.3%) affiliated with type 2 antibiotic monotherapy, and 12 (0.7%) affiliated with type 3 antibiotic monotherapy. There were 83 cases (0.8%) of AAE in the type 1 + 3 combination therapy group and one case (0.1%) in the type 2 + 3 combination therapy group.

**Figure 2:**
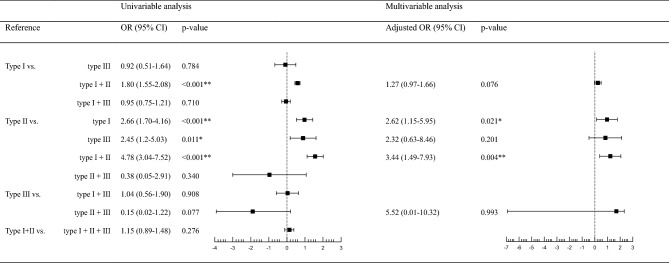
Association between exposure to the antibiotics and AAE incidence. Multivariable analysis was performed with logistic regression analysis adjusted by age, sex, serum white blood cell, erythrocyte sedimentation rate, c-reactive protein, and glomerular filtration rate (*p < 0.05, **p < 0.01). The values of the Odds ratios in the forest plot were the transformed values by natural log.

Categorized groups of monotherapy and combination therapy were compared to discern the incidence of AAE (Fig. 2). The type 1 monotherapy group showed a significantly higher incidence of AAE compared to the type 2 monotherapy group, with an odds ratio (OR) of 2.66 (confidence interval [CI] 1.7–4.16; *P* < 0.001).

In multi-variable analysis, cases receiving type 1 antibiotics had a higher OR for AAE compared to those receiving type 2 antibiotics after adjusting for age, sex, white blood cell count (WBC), ESR and CRP levels, and GFR (adjusted OR, 2.62; CI 1.15–5.95; *P* = 0.021). However, the type 1 monotherapy group did not show any significant difference in comparison with the type 3 monotherapy group (OR, 0.92; CI 0.51–1.64; *P* = 0.784). Compared to the type 2 monotherapy group, the type 3 monotherapy group showed a greater occurrence of AAE (OR, 2.45; CI 1.20–5.03; *P* = 0.011), although there was no significant difference after adjustment for covariables (adjusted OR, 2.32; CI 0.63–8.46; *P* = 0.201).

Combined use of type 1 + 2 antibiotics was associated with a 2.16-fold higher incidence of AAE compared to that in the type 1 monotherapy group (OR, 1.80; CI 1.55–2.08; *P* < 0.001). This comparison failed to demonstrate significant difference after adjustments for covariables, although trends were observed (adjusted OR, 1.27; CI 0.97–1.66; *P* = 0.076). The incidence of AAE in the type 1 + 2 combination therapy group was significantly higher compared to the type 2 monotherapy group, and significance remained with covariate adjustment (OR, 4.78; CI 3.04–7.52; *P* < 0.001 vs. adjusted OR, 3.44; CI 1.49–7.93; *P* = 0.004).

The type 1 + 3 combination therapy group did not show significant difference compared to the type 1 monotherapy group (OR, 0.95; CI 0.75–1.21; *P* = 0.710) or the type 3 monotherapy group (OR, 1.04; CI 0.56–1.90; *P* = 0.908) regarding the development of AAE. There was no significant difference between the type 2 monotherapy group and the type 2 + 3 combination therapy group (OR, 0.38; CI 0.05–2.91; *P* = 0.340) or between the type 3 monotherapy and type 2 + 3 combination therapy groups (OR, 0.15; CI 0.02–1.22; *P* = 0.077). The marginal trend between the type 3 monotherapy group and type 2 + 3 combination therapy group was attenuated by adjustments of confounding variables (adjusted OR, 5.52; CI 0.01–10.32; *P* = 0.993). The combination of types 1–3 antibiotics did not lead to any significant difference compared to the combination of type 1 + 2 antibiotics (OR, 1.15; CI 0.89–1.48; *P* = 0.276).

### Association between GFR and AAE

**Figure 3:**
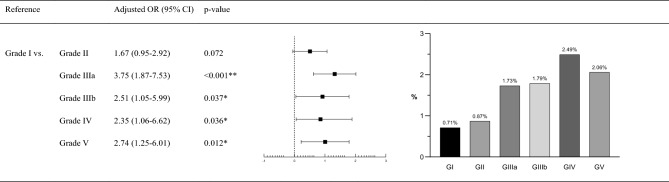
Association between GFR and AAE incidence according to the antibiotics, and the incidence of AAE in combination therapy of type 1 + 2 antibiotics according to GFR. Calculated GFR is categorized into 6 groups by the range of the GFR value; Grade 1 (normal or high, ≥ 90 mL/min/1.73 m^2^), Grade 2 (mildly decreased, 60–89 mL/min/1.73 m^2^), Grade 3a (mild to moderately decreased, 45–59 mL/min/1.73 m^2^), Grade 3b (moderately to severely decreased, 30–44 mL/min/1.73 m^2^), Grade 4 (severely decreased, 15–29 mL/min/1.73 m^2^), and Grade 5 (kidney failure, < 15 mL/min/1.73 m^2^). Multivariable analysis was performed with logistic regression analysis adjusted by age, sex, serum WBC, ESR, CRP, and GFR (*p < 0.05, **p < 0.01). The values of the ORs in the forest plot were the transformed values by natural log. *AAE* Antibiotic-associated encephalopathy. GFR: calculated using 2021 CKD-EPI (chronic kidney disease-Epidemiology Collaboration) Creatinine.

The GFR was categorized into six groups according to the range of GFR value: G1 (normal or high, ≥ 90 mL/min/1.73 m^2^), G2 (mildly decreased, 60–89 mL/min/1.73 m^2^), G3a (mild to moderately decreased, 45–59 mL/min/1.73 m^2^), G3b (moderately to severely decreased, 30–44 mL/min/1.73 m^2^), G4 (severely decreased, 15–29 mL/min/1.73 m^2^), and G5 (kidney failure, < 15 mL/min/1.73 m^2^)^15^. The degree of renal insufficiency was analyzed for the type 1 + 2 combination therapy group, which had the highest OR for AAE among groups. As the GFR level decreased, the incidence of AAE increased significantly, as shown in Fig. 3. G3, G4, and G5 had significantly higher ORs for AAE compared to G1.

### EEG patterns according to antibiotics used

In EEG analysis of admission cases with AAE where EEG was performed, although periodic discharges (PDs) and triphasic morphology appeared only in the admission group receiving type 1 antibiotics either as monotherapy or as part of combination therapy (Table 2), statistical significance was not observed in either pattern in a comparison study with the other admission groups (OR, 2.31; CI 0.138–38.5; *P* = 0.290 for PDs, OR, 2.39; CI 0.14–39.8; *P* = 0.282 for triphasic morphology pattern). No significant difference was observed in the rhythmic delta activity (RDA) patterns of type 1 and non–type 1 antibiotics admission groups (OR, 1.54; CI 0.20–11.5; *P* = 0.672). Detection of the spike-and-wave or sharp-and-wave (SW) pattern was significantly more common in the triple combination therapy group (OR, 2.91; CI 1.66–5.12; *P* < 0.001), but the significance decreased to a trend after adjusting for age, sex, WBC, ESR and CRP levels, and GFR (adjusted OR, 2.65; CI 0.97–7.25; *P* = 0.057). All of the sharp waves observed on EEG were of interictal nature, and clinically, they were not associated with either convulsive status epilepticus or non-convulsive electrical EEG seizures.

**Table 2:** Electroencephalogram patterns in admission cases with antibiotic-associated encephalopathy.

	PD without triphasic wave	PD with triphasic wave	SW	RDA
Type 1	14 (3.1)	10 (2.2)	45 (9.8)	23 (5)
Type 2	0 (0)	0 (0)	1 (5)	0 (0)
Type 3	0 (0)	0 (0)	1 (8.3)	1 (8.3)
Type 1&2	9 (3)	10 (3.4)	32 (10.7)	11 (3.7)
Type 1&3	2 (2.4)	5 (6)	9 (10.8)	4 (4.8)
Type 2&3	0 (0)	0 (0)	0 (0)	0 (0)
Type 1,2, and 3	5 (6.5)	6 (7.8)	19 (24.7)	4 (5.2)

Values are the number of patients (%).

*PD* Periodic discharges, *SW* Spike-and-wave or sharp-and-wave, *RDA* Rhythmic delta activity.

## Discussion

The growing number of cases of AAE has drawn attention to the neurotoxic side effects of antibiotic use^16,17^. The effect of antibiotics by type on the incidence of AAE was examined in this study. In the comparison of monotherapy groups, type 1 antibiotics was associated with a significantly higher incidence of AAE compared to type 2 antibiotics. Combination use of type 1 + 2 antibiotics tended to lead to more prevalent AAE compared to 2 monotherapy. The tendency for AAE increased significantly as renal function decreased in the analysis of type 1 + 2 combination therapy. G3, G4, and G5 showed significantly higher incidence rates of AAE compared to G1. Finally, the EEG study revealed a relationship with PDs pattern including triphasic morphology when using type 1 antibiotics either as monotherapy or as part of combination therapy. Epileptiform discharges were more likely to be detected with combined use of all three types of antibiotics.

Prior studies on AAE have focused on individual antibiotic effects on neurological dysfunction by clinical manifestations and patient susceptibility depending on the risk factors of older age, comorbidities, and metabolic dysfunction^5,7,10–12,14,18,19^. To our knowledge, this is the first large study to incorporate a wide range of antibiotics utilized for cases ranging in severity from minor to severe in a tertiary hospital setting.

Presenting a novel perspective of AAE, this study categorized antibiotics by type in both monotherapy and combination therapy protocols for comparison of AAE incidence rates. Combination use of type 1 + 2 antibiotics tended to lead to more prevalent AAE compared to type 2 monotherapy. Based on the unique actions on the CNS associated with each type of antibiotic, combination use is expected to increase the chance of encephalopathy. Type 1 antibiotics are thought to cause encephalopathy through disruption of inhibitory synaptic transmission leading to excitotoxicity through ligand-gated ion channel ɤ-aminobutyric acid class A receptor (GABA_A_R) binding^5,9,11,12,14,18^. β-lactams bind GABA_A_R either competitively or non-competitively and inhibit intracellular influx of chloride, impeding inhibitory postsynaptic potential. The type 2 antibiotic group either stimulates GABA receptors in the brain, activating epileptic activity, or affects N-methyl-D-aspartate (NMDA) glutamate receptors in a concentration-dependent manner, decreasing the seizure threshold^9,20^.

Our results support previous reports on renal insufficiency attributed to the neurotoxicity associated with in use of antibiotics. The prevalence of antibiotic-induced neurotoxicity incidence in chronic renal insufficiency patients is reported to be in the range of 3.0–16.6%^21^. The free antibiotic drug concentration increases as a result of the decreased kidney clearance rate and an increasing lack of serum protein for binding due to proteinuria^9,22,23^. The accumulation of toxins in serum also inhibits active transportation of antibiotics from the cerebrospinal fluid across the blood–brain barrier^9,22,23^. In addition, decreased protein levels reduce the integrity of the blood–brain barrier by decreasing protein glycation and carbamylation, enhancing entry of the antibiotics into the CNS^9,22,23^.

In the study of EEG findings associated with AAE, the prevalence of epileptiform discharges ranged from 0 to 24.7% by the classes of antibiotics, and the PDs and triphasic morphology appeared only in the group receiving type 1 antibiotics either as monotherapy or as part of combination therapy. Previously, epileptogenic properties of antibiotics have been reported^20,23^. Disturbance in GABAergic transmission either by direct or indirect antagonistic mechanisms or by inhibiting the synthesis of GABA triggers the activation of epileptic activity^20,23^. In addition, change in NMDA receptor to decrease the seizure threshold is another epileptogenic property^20,23^. The larger is the number of antibiotic types added, the greater is the resulting damage, supporting the development of epileptiform discharges as an augmentation effect on the CNS.

While this study has strength in its large number of cases and comparisons among antibiotics, some limitations should be considered. As cases were selected based on admission events instead of patients, repeated counts were inevitable, which may have lowered the incidence of AAE. Nonetheless, admission events instead of patient count were analyzed because of the many subjects with multiple admissions with different antibiotic treatment types. In addition, another limitation of our study is that we did not consider disease entity but only antibiotics type despite our attempts to control for the confounding effects of infectious status with laboratory variables such as WBC count, ESR and CRP levels, and renal function.

One significant limitation of this study is the potential confounding factor of severe infectious disease states, which may independently induce neurological symptoms such as myoclonus or psychotic episodes, particularly in relation to delirium. The use of combination antibiotics therapy indicates a broader spectrum of pathogens to address, which indirectly suggests a more severe infectious state. In patients with sepsis, circulating cytokines, either directly or through oxidative stress, are related to neurotoxicity^24–26^. Encephalopathy is expected to occur more frequently in patients with severe infection, as the infection state is presumed to influence the brain^27–29^. This is supported by generalized PDs with triphasic morphology in critically ill patients due to disinhibition of excitatory pyramidal cells from either a synaptic failure of interneurons or impaired excitation of inhibitory interneurons^29^. Finally, drugs not categorized as type 1 or type 2 antibiotics were designated as type 3 antibiotics, despite possessing distinct mechanisms of action. While the utilization of type 3 antibiotics, whether in monotherapy or combination therapy, constituted only 17% of this study cases, it represents a noteworthy limitation in this study.

In comparison to type 2 antibiotics including sulfonamides, fluoroquinolones, and macrolides, the type 1 β-lactam antibiotics, whether used alone or in combination with type 2 antibiotics, was associated with a heightened incidence of AAE and the presence of PDs or triphasic waves on EEG. More comprehensive studies on encephalopathy arising from antibiotics use should be performed for further understanding of AAE.

## Materials and methods

### Ethical approval

All aspects of this retrospective study were approved and the requirement for informed consent was formally waived by the Institutional Review Board of The Catholic University of Korea (XC22WADI0015).

### Data sources

Consecutive admissions to the hospitals involving antibiotics for treatment of infectious disease between June 1, 2018, and January 1, 2021, were identified from six tertiary referral university hospitals (Bucheon St. Mary’s Hospital, Incheon St. Mary’s Hospital, St. Vincent’s Hospital, Seoul St. Mary’s Hospital, Uijeongbu St. Mary’s Hospital, and Yeouido St. Mary’s Hospital). We used data from the Clinical Data Warehouse database, which is a large, integrated, harmonized database of these medical centers belonging to The Catholic University of Korea, College of Medicine, Seoul, Korea. The information in this database has been collected from electronic medical records and order communication systems since April 1997, with the aforementioned hospitals commonly sharing platforms.

### Study cohort

In this retrospective cohort study, we used data from the Clinical Data Warehouse (CDW) database, which is a large, integrated, harmonized database of five tertiary referral medical centers belonging to The Catholic University of Korea, College of Medicine, Seoul, Korea. The information in this database has been collected from electronic medical records (EMR) and order communication system (OCS) since April 1997, the platforms of which are commonly shared by the five hospitals. Briefly, the relational CDW database is composed of a demographics database; a diagnosis database including participant age and date at diagnosis; an admission database including admission dates, discharge dates, and information on inpatients and outpatients; a consultation database; a prescription database including prescribed medications and ordered laboratory tests; a laboratory database with results of ordered tests and date of the test; and a histology database. Data extraction is performed after anonymization, but researchers are able to access the EMR and OCS of each participant without identifying participant personal information. In this study, conducted by a team of neurologists using the CDW database, the investigation focused on identifying encephalopathy, myoclonus, and/or seizure events, that were either reversed upon discontinuation of implicated antibiotic agents or conclusively attributed to antibiotics, as confirmed through consultations with neurologists (Fig. 1).

Consultations for conditions other than encephalopathy, myoclonus, and/or seizure were excluded, and patients with encephalopathy caused by primary brain disorders including traumatic injury, encephalitis/meningitis, structural lesions such as tumor or metastatic disease, arterial or venous stroke, mass effect/increased intracranial pressure, degenerative disease, and epilepsy or patients with secondary brain disorders due to renal/hepatic dysfunction, electrolyte abnormalities, hyper- or hypoglycemia, endocrine dysfunction, nutritional deficiencies, CNS toxins, or anoxia/hypoxia/hypercapnia were excluded. Encephalopathy was considered when there was any alteration in mental status affecting cognition or level of arousal by global brain insult^1^, with possible signs and symptoms including (1) delirium/mild encephalopathy, in which patients may be agitated, confused, inattentive, drowsy, and/or have perceptual problems and hallucinations, and (2) stupor or coma, where patients have a depressed level of consciousness but are arousable with vigorous stimulation or unresponsive to external stimuli.

Medical records were reviewed for age, sex, primary diagnosis, and antibiotics used during admission. Initial measurements of serum WBC, inflammatory markers of ESR and CRP level, blood urea nitrogen, serum creatinine level, and GFR calculated using the 2021 Chronic Kidney Disease–Epidemiology Collaboration creatinine concentration were obtained^30^. The calculated GFR was categorized into six groups according to the range of GFR value: G1 (normal or high, ≥ 90 mL/min/1.73 m^2^), G2 (mildly decreased, 60–89 mL/min/1.73 m^2^), G3a (mild to moderately decreased, 45–59 mL/min/1.73 m^2^), G3b (moderately to severely decreased, 30–44 mL/min/1.73 m^2^), G4 (severely decreased, 15–29 mL/min/1.73 m^2^), and G5 (kidney failure, < 15 mL/min/1.73 m^2^)^15^.

### Classification of antibiotics

Bhattacharyya et al. performed extensive analysis regarding AAE and found three distinct clinical subtypes of AAE, caused by different antibiotics, and based on unique pathophysiologic mechanisms^9^. The classification is based on antibiotic-induced pathophysiologic mechanisms of encephalopathy and the resulting characteristic phenotypes of clinical symptoms, temporal evolution, and laboratory abnormalities. Type 1 AAE is characterized by myoclonus or seizures, abnormal EEG findings, and normal MRI results. This phenotype is mostly caused by β-lactam antibiotics, including penicillin and cephalosporins, through the mechanism of disruption of inhibitory synaptic transmission leading to excitotoxicity. Type 2 AAE is characterized by psychosis and abnormal EEG results and may be caused by sulfonamides, fluoroquinolones, and macrolides via perturbations of the D2 dopamine and NMDA glutamate receptors. Type 3 AAE is diagnosed when there is cerebellar dysfunction with abnormal MRI results caused by metronidazole. Its mechanism is related to free radical formation and altered thiamine metabolism.

Here, we classified antibiotics according to type 1 and 2 AAE phenotypes. We classified any that did not fit these classes into type 3 AAEs in addition to metronidazole because the type 3 AAE of Bhattacharya’s classification includes only metronidazole. Therefore, we categorized antibiotics into 3 groups, as follows: (1) type I antibiotics included Amoxicillin, Ampicillin, Cefazolin, Cefepime, Cefobactam, Cefoperazone, Cefotaxime, Cefotetan, Cefoxitin, Ceftazidime, Ceftizoxime, Ceftriaxone, Cefuroxime, doripenem, Ertapenem, Flomoxef, Imipenem, Meropenem, Nafcillin, Penicillin, Piperacillin, and Prepenem; (2) type 2 antibiotics included Azithromycin, Ciprofloxacin, Clarithromycin, Clindamycin, Cotrimoxazole, Gemifloxacin, levofloxacin, moxifioxacin, Roxithromycin, and Tosufloxacin; and (3) type 3 antibiotics included Cycloserine, Dapsone, Ethambutol, Isoniazid, Metronidazole, pyrazinamide, rifampicin (Supplementary Table 1).

Based on these groups, seven combinations were identified: monotherapy with any one of the antibiotics; combined therapy of type 1 + 2, type 1 + 3, or type 2 + 3; and triple therapy using types 1 + 2 + 3 (Fig. 1).

### EEG recordings and analysis

We gathered standard EEG recordings with pad electrodes depending on the clinical situation according to the International 10–20 system for electrode placement with additional subtemporal electrodes (T1/T2). The minimal EEG duration was 30 min.

Obtained EEG patterns were classified according to the American Clinical Neurophysiology Society Standardized Critical Care EEG Terminology criteria^31^. The prevalent pattern of EEG was classified into PDs with subcategories of triphasic morphology; RDA; and SW, for which showed more than or equal to abundant > 50% predominance in each record. If EEG results did not conform to any of the above patterns, including diffuse slow-wave activity or background delta or theta slow activity, it was classified into an “other EEG pattern” subgroup.

### Statistical analysis

Statistical analyses were performed using SPSS for Windows version 28.0 (IBM Corporation, Armonk, NY, USA). Pearson’s chi-square test and Fisher’s exact test were used for categorical variable comparison, and analysis of variance was used for comparison of continuous variables. Values are expressed as mean ± standard deviation and as number of patients with percentage. The ORs for AAE according to types of antibiotics used were calculated using logistic multi-variable regression analysis adjusted for age, sex, serum levels of ESR and CRP, WBC count (to account for the potential confounding effect of infection on encephalopathy), and GFR (to address the potential confounding impact of antibiotic drug elimination).

Variables displaying significant differences at P < 0.1 among the groups were incorporated as covariates in the logistic multivariable regression model. In two-tailed analysis, *P* < 0.05 was considered statistically significant.

### Ethical standards

All procedures performed in studies involving human participants were in accordance with the ethical standards of the institutional committee and the 1964 Declaration of Helsinki and its later amendments or comparable ethical standards.

### Supplementary Information

Supplementary Table 1.

## Data Availability

The de-identified data supporting the findings of this study are available upon reasonable request to the corresponding author.
