# Supplementary material for: The incidence and predictors of antibiotic-associated encephalopathy: a multicenter hospital-based study
Source: Sci Rep. 2024 Apr 16;14:8747. doi: 10.1038/s41598-024-59555-w (PMC11021399; doi:10.1038/s41598-024-59555-w)
Supplement: Supplementary file 1 — Supplementary Table 1. [file 41598_2024_59555_MOESM1_ESM.docx]

| Supplementary table. Types of antibiotics used and their proportions by each group. | | | |
| --- | --- | --- | --- |
| Subgroup | Antibiotics used | Number of admission cases | Per cent of admission cases (%) |
| Type I antibiotics | Ceftriaxone | 9270 | 17.1 |
|  | Ampicillin | 7403 | 13.6 |
|  | Cefazolin | 5323 | 9.8 |
|  | Cefotetan | 5241 | 9.7 |
|  | piperacillin | 4563 | 8.4 |
|  | Flomoxef | 3852 | 7.1 |
|  | Cefobactam | 3436 | 6.3 |
|  | Ceftizoxime | 3087 | 5.7 |
|  | Cefepime | 2797 | 5.2 |
|  | Cefoxitin | 2499 | 4.6 |
|  | Cefoperazone | 2153 | 4 |
|  | Amoxicillin | 1597 | 2.9 |
|  | Cefotaxime | 1038 | 1.9 |
|  | Meropenem | 617 | 1.1 |
|  | Cefuroxime | 562 | 1 |
|  | Ceftazidime | 502 | 0.9 |
|  | Ertapenem | 130 | 0.2 |
|  | Doripenem | 73 | 0.1 |
|  | Imipenem | 64 | 0.1 |
|  | Penicillin | 42 | 0.1 |
|  | Prepenem | 9 | 0 |
|  | Nafcillin | 4 | 0 |
|  | Total | 54262 | 100 |
|  |  |  |  |
| Type II antibiotics | Ciprofloxacin | 2653 | 42.3 |
|  | Cotrimoxazole | 1676 | 26.7 |
|  | Levoflaxacin | 1397 | 22.3 |
|  | Moxifioxacin | 261 | 4.2 |
|  | Clarithromycin | 117 | 1.9 |
|  | Roxithromycin | 105 | 1.7 |
|  | Clindamycin | 23 | 0.4 |
|  | Tosufloxacin | 22 | 0.4 |
|  | Azithromycin | 18 | 0.3 |
|  | Gemifloxacin | 3 | 0.05 |
|  | Total | 6275 | 100 |
|  |  |  |  |
| Type III antibiotics | Metronidazole | 1307 | 84.7 |
|  | Ethambutol | 79 | 5.1 |
|  | Rifampicin | 57 | 3.7 |
|  | Pyrazinamide | 43 | 2.8 |
|  | Isoniazid | 39 | 2.5 |
|  | Dapsone | 14 | 0.9 |
|  | Cycloserine | 4 | 0.3 |
|  | Total | 1543 | 100 |
|  |  |  |  |
|  |  |  |  |
| Type I + II antibiotics | Ceftriaxone + Erythromycin | 1820 | 9.2 |
|  | Ceftriaxone + Ciprofloxacin | 1485 | 7.5 |
|  | Piperacillin + Levofloxacin | 1428 | 7.2 |
|  | Ceftriaxone + Levofloxacin | 1374 | 6.9 |
|  | Cefepime + Ciprofloxacin | 840 | 4.2 |
|  | Cefepime + Levofloxacin | 756 | 3.8 |
|  | Cefazolin + Ciprofloxacin | 664 | 3.4 |
|  | Piperacillin + Ciprofloxacin | 643 | 3.2 |
|  | Ceftriaxone + Clindamycin | 612 | 3.1 |
|  | Ceftizoxime + Erythromycin | 546 | 2.8 |
|  | Ceftriaxone + Cotrimoxazole | 522 | 2.6 |
|  | Cefobactam + Ciprofloxacin | 446 | 2.3 |
|  | Ceftizoxime + Levofloxacin | 402 | 2.0 |
|  | Cefuroxime + Erythromycin | 371 | 1.9 |
|  | Cefepime + Cotrimoxazole | 364 | 1.8 |
|  | Eeftriaxone + Moxifloxacin | 353 | 1.8 |
|  | Piperacillin + Moxifloxacin | 352 | 1.8 |
|  | Cefepime + Moxifloxacin | 340 | 1.7 |
|  | Cefotaxime + Ciprofloxacin | 338 | 1.7 |
|  | Ceftizoxime + Ciprofloxacin | 295 | 1.5 |
|  | Flomoxef + Cotrimoxazole | 294 | 1.5 |
|  | Amoxicillin + Erythromycin | 260 | 1.3 |
|  | Amoxicillin + Clindamycin | 253 | 1.3 |
|  | Amoxicillin + Ciprofloxacin | 237 | 1.2 |
|  | Ceftazidime + Levofloxacin | 236 | 1.2 |
|  | Meropenem + Ciprofloxacin | 214 | 1.1 |
|  | Cefuroxime + Ciprofloxacin | 209 | 1.1 |
|  | Certazidime + Ciprofloxacin | 176 | 0.9 |
|  | Piperacillin + Erythromycin | 176 | 0.9 |
|  | Piperacillin + Cotrimoxazole | 171 | 0.9 |
|  | Piperacillin + Clindamycin | 165 | 0.8 |
|  | Cefepime + Erythromycin | 161 | 0.8 |
|  | Meropenem + Levofloxacin | 160 | 0.8 |
|  | Flomoxef + Levofloxacin | 148 | 0.7 |
|  | Cefoperazone + Ciprofloxacin | 144 | 0.7 |
|  | Ceftizoxime + Clindamycin | 137 | 0.7 |
|  | Cefazolin + Levofloxacin | 126 | 0.6 |
|  | Amoxicillin + Levofloxacin | 120 | 0.6 |
|  | Cefobactam + Levofloxacin | 119 | 0.6 |
|  | Cefazolin + Cotrimoxazole | 113 | 0.6 |
|  | Cefotetan + Ciprofloxacin | 101 | 0.5 |
|  | Flomoxef + Erythromycin | 95 | 0.5 |
|  | Ampicillin + Ciprofloxacin | 92 | 0.5 |
|  | Meropenem + Cotrimoxazole | 88 | 0.4 |
|  | Cefepime + Clindamycin | 86 | 0.4 |
|  | Cefobactam + Moxifloxacin | 81 | 0.4 |
|  | Ceftazidime + Cotrimoxazole | 81 | 0.4 |
|  | Flomoxef + Ciprofloxacin | 81 | 0.4 |
|  | Amoxicillin + Cotrimoxazole | 79 | 0.4 |
|  | Cefazolin + Clindamycin | 70 | 0.4 |
|  | Cefobactam + Erythromycin | 62 | 0.3 |
|  | Cefoperazone + Levofloxacin | 62 | 0.3 |
|  | Ceftriaxone + Azithromycin | 59 | 0.3 |
|  | Ampicillin + Cotrimoxazole | 53 | 0.3 |
|  | Cefoxitin + Ciprofloxacin | 51 | 0.3 |
|  | Cefotetan + Levofloxacin | 46 | 0.2 |
|  | Ceftazidime + Moxifloxacin | 43 | 0.2 |
|  | Ampicillin + Levofloxacin | 40 | 0.2 |
|  | Meropenem + Moxifloxacin | 40 | 0.2 |
|  | Cefazolin + Erythromycin | 37 | 0.2 |
|  | Ceftazidime + Erythromycin | 36 | 0.2 |
|  | Meropenem + Erythromycin | 36 | 0.2 |
|  | Cefobactam + Clindamycin | 34 | 0.2 |
|  | Cefotaxime + Erythromycin | 33 | 0.2 |
|  | Ampicillin + Erythromycin | 30 | 0.2 |
|  | Cefotetan + Cotrimoxazole | 30 | 0.2 |
|  | Cefuroxime + Levofloxacin | 29 | 0.1 |
|  | Imipenem + Levofloxacin | 29 | 0.1 |
|  | Ertapenem + Ciprofloxacin | 28 | 0.1 |
|  | Amoxicillin + Moxifloxacin | 27 | 0.1 |
|  | Cefoperazone + Moxifloxacin | 27 | 0.1 |
|  | Ceftizoxime + Moxifloxacin | 27 | 0.1 |
|  | Flomoxef + Moxifloxacin | 25 | 0.1 |
|  | Imipenem + Ciprofloxacin | 25 | 0.1 |
|  | Cefoperazone + Cotrimoxazole | 24 | 0.1 |
|  | Piperacillin + Azithromycin | 24 | 0.1 |
|  | Cefuroxime + Cotrimoxazole | 23 | 0.1 |
|  | Cefepime + Azithromycin | 19 | 0.1 |
|  | Ceftizoxime + Cotrimoxazole | 19 | 0.1 |
|  | Cefobactam + Cotrimoxazole | 17 | 0.1 |
|  | Cefoperazone + Erythromycin | 17 | 0.1 |
|  | Imipenem + Cotrimoxazole | 17 | 0.1 |
|  | Cefotaxime + Cotrimoxazole | 15 | 0.1 |
|  | Ceftazidime + Clindamycin | 15 | 0.1 |
|  | Doripenem + Levofloxacin | 15 | 0.1 |
|  | Cefotaxime + Levofloxacin | 14 | 0.1 |
|  | Ertapenem + Cotrimoxazole | 13 | 0.1 |
|  | Cefotaxime + Clindamycin | 12 | 0.1 |
|  | Cefoxitin + Erythromycin | 11 | 0.1 |
|  | Ceroxitin+ Levofloxacin | 11 | 0.1 |
|  | Cefuroxime + Clindamycin | 11 | 0.1 |
|  | Imipenem + Erythromycin | 11 | 0.1 |
|  | Cefazolin+Moxifloxacin | 10 | 0.1 |
|  | Cefoxitin+Cotrimoxazole | 10 | 0.1 |
|  | Ertapenem +Levofloxacin | 10 | 0.1 |
|  | Ampicillin + Moxifloxacin | 9 | 0.0 |
|  | Cefoperazone + Clindamycin | 9 | 0.0 |
|  | Cerotetan + Moxifloxacin | 9 | 0.0 |
|  | Imipenem + Moxifloxacin | 9 | 0.0 |
|  | Cefotetan + Erythromycin | 8 | 0.0 |
|  | Amoxicillin + Azithromycin | 7 | 0.0 |
|  | Meropenem + Clindamycin | 7 | 0.0 |
|  | Cefotetan + Clindamycin | 6 | 0.0 |
|  | Doripenem + Ciprofloxacin | 5 | 0.0 |
|  | Doripenem + Moxifloxacin | 5 | 0.0 |
|  | Penicillin + Ciprofloxacin | 5 | 0.0 |
|  | Flomoxef + Tosufloxacin | 4 | 0.0 |
|  | Meropenem + Azithromycin | 4 | 0.0 |
|  | Ampicillin + Azithromycin | 3 | 0.0 |
|  | Ampicillin + Clindamycin | 3 | 0.0 |
|  | Cefotaxime+Moxifloxacin | 3 | 0.0 |
|  | Ceftriaxone + Tosufloxacin | 3 | 0.0 |
|  | Cefuroxime + Moxifloxacin | 3 | 0.0 |
|  | Ertapenem + Moxifloxacin | 3 | 0.0 |
|  | Flomoxer + Clindamycin | 3 | 0.0 |
|  | Penicillin + Erythromycin | 3 | 0.0 |
|  | Penicillin + Levofloxacin | 3 | 0.0 |
|  | Cetoperazone+Azithromycin | 2 | 0.0 |
|  | Doripenem+Clindamycin | 2 | 0.0 |
|  | Doripenem +Erythromycin | 2 | 0.0 |
|  | Imipenem + Azithromycin | 2 | 0.0 |
|  | Nafcillin + Ciprofloxacin | 2 | 0.0 |
|  | Nafcillin + Levofloxacin | 2 | 0.0 |
|  | Penicillin + Clindamycin | 2 | 0.0 |
|  | Penicillin + Cotrimoxazole | 2 | 0.0 |
|  | Cefazolin + Tosufloxacin | 1 | 0.0 |
|  | Cefobactam + Azithromycin | 1 | 0.0 |
|  | Cefoperazone + Tosufloxacin | 1 | 0.0 |
|  | Cefotetan + Azithromycin | 1 | 0.0 |
|  | Cefoxitin + Azithromycin | 1 | 0.0 |
|  | Cefoxitin + Moxifloxacin | 1 | 0.0 |
|  | Ceftazidime + Azithromycin | 1 | 0.0 |
|  | Ceftizoxime + Azithromycin | 1 | 0.0 |
|  | Doripenem + Azithromycin | 1 | 0.0 |
|  | Ertapenem + Azithromycin | 1 | 0.0 |
|  | Ertapenem + Erythromycin | 1 | 0.0 |
|  | Flomoxef + Azithromycin | 1 | 0.0 |
|  | Imipenem + Clindamycin | 1 | 0.0 |
|  | Total | 19794 | 100 |
|  |  |  |  |
| Type I + III | Ceftriaxone + Metronidazole | 4835 | 46.9 |
|  | Cefotetan + Metronidazole | 738 | 7.2 |
|  | cefoperazone + Metronidazole | 701 | 6.8 |
|  | Cefazolin + Metronidazole | 669 | 6.5 |
|  | Cefobactam + Metronidazole | 592 | 5.7 |
|  | Piperacillin + Metronidazole | 537 | 5.2 |
|  | Amoxicillin + Metronidazole | 381 | 3.7 |
|  | Cefepime + Metronidazole | 293 | 2.8 |
|  | Ceftizoxime + Metronidazole | 258 | 2.5 |
|  | Flomoxef + Metronidazole | 189 | 1.8 |
|  | Ceftizoxime + Ethambutol | 127 | 1.2 |
|  | Ceftriaxone + Ethambutol | 122 | 1.2 |
|  | Meropenem + Metronidazole | 106 | 1.0 |
|  | Ampicillin + Metronidazole | 98 | 1.0 |
|  | Cefotaxime + Metronidazole | 97 | 0.9 |
|  | Ceftazidime + Metronidazole | 94 | 0.9 |
|  | Cefoxitin + Metronidazole | 93 | 0.9 |
|  | Piperacillin + Ethambutol | 43 | 0.4 |
|  | Cefuroxime + Metronidazole | 39 | 0.4 |
|  | Cefepime + Ethambutol | 18 | 0.2 |
|  | Ceftriaxone + Rifampicin | 16 | 0.2 |
|  | Cefuroxime + Ethambutol | 16 | 0.2 |
|  | Flomoxef + Ethambutol | 16 | 0.2 |
|  | Imipenem + Metronidazole | 16 | 0.2 |
|  | Ceftriaxone + Isoniazid | 15 | 0.1 |
|  | Cefazolin + Rifampicin | 12 | 0.1 |
|  | Ceftriaxone + Pyrazinamide | 12 | 0.1 |
|  | Cefazolin + Ethambutol | 11 | 0.1 |
|  | Cetobactam + Ethambutol | 11 | 0.1 |
|  | Doripenem + Metronidazole | 11 | 0.1 |
|  | Ertapenem + Metronidazole | 9 | 0.1 |
|  | Meropenem + Ethambutol | 9 | 0.1 |
|  | Amoxicillin + Ethambutol | 8 | 0.1 |
|  | Piperacillin + Rifampicin | 8 | 0.1 |
|  | Cefazolin + Isoniazid | 7 | 0.1 |
|  | Cefepime + Rifampicin | 7 | 0.1 |
|  | Meropenem + Rifampicin | 6 | 0.1 |
|  | Ampicillin + Ethambutol | 5 | 0.0 |
|  | Cefotetan + Ethambutol | 5 | 0.0 |
|  | Cetepime + Pyrazinamide | 4 | 0.0 |
|  | Cefotetan + Rifampicin | 4 | 0.0 |
|  | Ceftazidime + Ethambutol | 4 | 0.0 |
|  | Ceftazidime + Rifampicin | 4 | 0.0 |
|  | Cettizoxime + Pyrazinamide | 4 | 0.0 |
|  | Amoxicillin + Rifampicin | 3 | 0.0 |
|  | Cerepime + Isoniazia | 3 | 0.0 |
|  | Cefobactam + Pyrazinamide | 3 | 0.0 |
|  | Cefoperazone + Ethambutol | 3 | 0.0 |
|  | Cefoxitin + Ethambutol | 3 | 0.0 |
|  | Flomoxef + Isoniazid | 3 | 0.0 |
|  | Penicillin + Metronidazole | 3 | 0.0 |
|  | Amoxicillin + Pyrazinamide | 2 | 0.0 |
|  | Cefazolin + Pyrazinamide | 2 | 0.0 |
|  | Cefotetan + Isoniazid | 2 | 0.0 |
|  | Ceftazidime + Pyrazinamide | 2 | 0.0 |
|  | Ceftizoxime + Rifampicin | 2 | 0.0 |
|  | Ceftriaxone + Dapsone | 2 | 0.0 |
|  | Meropenem + Isoniazid | 2 | 0.0 |
|  | Piperacillin + Isoniazid | 2 | 0.0 |
|  | Piperacillin + Pyrazinamide | 2 | 0.0 |
|  | Amoxicillin + Isoniazid | 1 | 0.0 |
|  | Ampicillin + Rifampicin | 1 | 0.0 |
|  | Cefoperazone + Isoniazid | 1 | 0.0 |
|  | Cefoxitin + Pyrazinamide | 1 | 0.0 |
|  | Cefuroxime + Rifampicin | 1 | 0.0 |
|  | Doripenem + Ethambutol | 1 | 0.0 |
|  | Flomoxef + Pyrazinamide | 1 | 0.0 |
|  | Imipenem + Ethambutol | 1 | 0.0 |
|  | Meropenem + Pyrazinamide | 1 | 0.0 |
|  | Penicillin + Rifampicin | 1 | 0.0 |
|  | Piperacillin + Cycloserine | 1 | 0.0 |
|  | Total | 10300 |  |
|  |  |  |  |
|  | Ciprofloxacin + Metronidazole | 417 | 51.9 |
|  | Levofloxacin + Metronidazole | 90 | 11.2 |
|  | Cotrimoxazole + Isoniazid | 39 | 4.9 |
|  | Levofloxacin + Ethambutol | 38 | 4.7 |
|  | Erythromycin + Ethambutol | 31 | 3.9 |
|  | Moxifloxacin + Ethambutol | 31 | 3.9 |
|  | Levofloxacin + Cycloserine | 29 | 3.6 |
|  | Cotrimoxazole + Metronidazole | 18 | 2.2 |
|  | Levofloxacin + Isoniazid | 13 | 1.6 |
|  | Azithromycin + Ethambutol | 12 | 1.5 |
|  | Ciprofloxacin + Ethambutol | 12 | 1.5 |
|  | Moxifloxacin + Cycloserine | 10 | 1.2 |
|  | Levofloxacin + Rifampicin | 9 | 1.1 |
|  | Erythromycin + Metronidazole | 7 | 0.9 |
|  | Erythromycin + Rifampicin | 6 | 0.7 |
|  | Levofloxacin + Pyrazinamide | 6 | 0.7 |
|  | Moxifloxacin + Metronidazole | 6 | 0.7 |
|  | Moxifloxacin + Isoniazid | 5 | 0.6 |
|  | Cotrimoxazole + Rifampicin | 4 | 0.5 |
|  | Azithromycin + Metronidazole | 3 | 0.4 |
|  | Ciprofloxacin + Isoniazid | 3 | 0.4 |
|  | Cotrimoxazole + Ethambutol | 3 | 0.4 |
|  | Moxifloxacin + Rifampicin | 3 | 0.4 |
|  | Streptomycin + Cycloserine | 2 | 0.2 |
|  | Ciprofloxacin + Rifampicin | 1 | 0.1 |
|  | Clindamycin + Ethambutol | 1 | 0.1 |
|  | Clindamycin + Metronidazole | 1 | 0.1 |
|  | Clindamycin + Rifampicin | 1 | 0.1 |
|  | Erythromycin + Cycloserine | 1 | 0.1 |
|  | Erythromycin + Pyrazinamide | 1 | 0.1 |
|  | Moxifloxacin + Pyrazinamide | 1 | 0.1 |
|  | Total | 804 | 100 |
|  |  |  |  |
| Type I+II+III | Ceftriaxone + Ciprofloxacin + Metronidazole | 576 | 12.9 |
|  | Ceftriaxone + Levofloxacin + Metronidazole | 259 | 5.8 |
|  | Cefepime + Ciprofloxacin + Metronidazole | 239 | 5.4 |
|  | Piperacillin + Levofloxacin + Metronidazole | 168 | 3.8 |
|  | Cefobactam + Ciprofloxacin + Metronidazole | 162 | 3.6 |
|  | Cefazolin + Ciprofloxacin + Metronidazole | 148 | 3.3 |
|  | Piperacillin + Ciprofloxacin + Metronidazole | 139 | 3.1 |
|  | Cefazolin + Ciprofloxacin + Isoniazid | 132 | 3.0 |
|  | Cefepime + Levofloxacin + Metronidazole | 114 | 2.6 |
|  | Cefoperazone + Ciprofloxacin + Metronidazole | 91 | 2.0 |
|  | Ceftizoxime + Ciprofloxacin + Metronidazole | 89 | 2.0 |
|  | Ceftriaxone + Erythromycin + Metronidazole | 84 | 1.9 |
|  | Ceftriaxone + Clindamycin + Metronidazole | 76 | 1.7 |
|  | Ceftriaxone + Erythromycin + Ethambutol | 66 | 1.5 |
|  | Cefepime + Cotrimoxazole + Metronidazole | 65 | 1.5 |
|  | Ceftriaxone + Moxifloxacin + Metronidazole | 65 | 1.5 |
|  | Flomoxef + Cotrimoxazole + Isoniazid | 64 | 1.4 |
|  | Amoxicillin + Ciprofloxacin + Metronidazole | 60 | 1.3 |
|  | Ceftizoxime + Levofloxacin + Metronidazole | 57 | 1.3 |
|  | Ceftriaxone + Cotrimoxazole + Metronidazole | 57 | 1.3 |
|  | Piperacillin + Moxifloxacin + Metronidazole | 53 | 1.2 |
|  | Cefepime + Moxifloxacin + Metronidazole | 51 | 1.1 |
|  | Cefotetan + Ciprofloxacin + Metronidazole | 51 | 1.1 |
|  | Cefobactam + Levofloxacin + Metronidazole | 41 | 0.9 |
|  | Ceftazidime + Ciprofloxacin + Metronidazole | 40 | 0.9 |
|  | Ceftizoxime + Erythromycin + Ethambutol | 38 | 0.9 |
|  | Ceftazidime + Levofloxacin + Metronidazole | 35 | 0.8 |
|  | Piperacillin + Clindamycin + Metronidazole | 35 | 0.8 |
|  | Ceftriaxone + Clindamycin + Ethambutol | 34 | 0.8 |
|  | Meropenem + Ciprofoxacin + Metronidazole | 33 | 0.7 |
|  | Cefepime + Erythromycin + Metronidazole | 30 | 0.7 |
|  | Meropenem + Levofloxacin + Metronidazole | 29 | 0.7 |
|  | Cefoperazone + Levofloxacin + Metronidazole | 27 | 0.6 |
|  | Cefobactam + Moxifloxacin + Metronidazole | 26 | 0.6 |
|  | Ceftizoxime + Erythromycin + Metronidazole | 26 | 0.6 |
|  | Cefazolin + Levofloxacin + Metronidazole | 25 | 0.6 |
|  | Ceftriaxone + Levofloxacin + Ethambutol | 25 | 0.6 |
|  | Flomoxef + Ciprofloxacin + Metronidazole | 25 | 0.6 |
|  | Amoxicillin + Erythromycin + Metronidazole | 24 | 0.5 |
|  | Piperacillin + Cotrimoxazole + Metronidazole | 24 | 0.5 |
|  | Ampicillin + Ciprofloxacin + Metronidazole | 23 | 0.5 |
|  | Cefuroxime + Erythromycin + Ethambutol | 23 | 0.5 |
|  | Cefepime + Cotrimoxazole + Isoniazid | 21 | 0.5 |
|  | Piperacillin + Erythromycin + Metronidazole | 21 | 0.5 |
|  | Ceftizoxime + Clindamycin + Metronidazole | 20 | 0.4 |
|  | Ceftriaxone + Cotrimoxazole + Isoniazid | 20 | 0.4 |
|  | Amoxicillin + Levofloxacin + Metronidazole | 19 | 0.4 |
|  | Cefepime + Ciprofloxacin + Isoniazid | 19 | 0.4 |
|  | Cefotaxime + Ciprofloxacin + Metronidazole | 19 | 0.4 |
|  | Piperacillin + Levofloxacin + Ethambutol | 19 | 0.4 |
|  | Amoxicillin + Clindamycin + Metronidazole | 18 | 0.4 |
|  | Ceftizoxime + Clindamycin + Ethambutol | 18 | 0.4 |
|  | Cefotetan + Levofloxacin + Metronidazole | 16 | 0.4 |
|  | Cefoxitin + Ciprofloxacin + Metronidazole | 16 | 0.4 |
|  | Cefazolin + Cotrimoxazole + Metronidazole | 15 | 0.3 |
|  | Amoxicillin + Cotrimoxazole + Metronidazole | 14 | 0.3 |
|  | Cefepime + Levofloxacin + Ethambutol | 14 | 0.3 |
|  | Ampicillin + Levofloxacin + Metronidazole | 13 | 0.3 |
|  | Ceftazidime + Cotrimoxazole + Metronidazole | 13 | 0.3 |
|  | Cefotaxime + Levofloxacin + Metronidazole | 12 | 0.3 |
|  | Flomoxef + Levofloxacin + Metronidazole | 12 | 0.3 |
|  | Piperacillin + Erythromycin + Ethambutol | 12 | 0.3 |
|  | Cefazolin + Clindamycin + Metronidazole | 11 | 0.2 |
|  | Cefazolin + Cotrimoxazole + Isoniazid | 11 | 0.2 |
|  | Flomoxef + Cotrimoxazole + Metronidazole | 11 | 0.2 |
|  | Ampicillin + Cotrimoxazole + Metronidazole | 10 | 0.2 |
|  | Cefuroxime + Ciprofloxacin + Metronidazole | 10 | 0.2 |
|  | Ceturoxime + Erythromycin + Metronidazole | 10 | 0.2 |
|  | Flomoxef + Erythromycin + Ethambutol | 10 | 0.2 |
|  | Piperacillin + Levofloxacin + Cycloserine | 10 | 0.2 |
|  | Piperacillin + Moxifloxacin + Ethambutol | 10 | 0.2 |
|  | Ampicillin + Erythromycin + Metronidazole | 9 | 0.2 |
|  | Cefepime + Clindamycin + Metronidazole | 9 | 0.2 |
|  | Cefepime + Moxifloxacin + Ethambutol | 9 | 0.2 |
|  | Cefobactam + Clindamycin + Metronidazole | 9 | 0.2 |
|  | Ceftriaxone + Azithromycin + Metronidazole | 9 | 0.2 |
|  | Ceftriaxone + Cotrimoxazole + Ethambutol | 9 | 0.2 |
|  | Cefazolin + Moxifloxacin + Metronidazole | 8 | 0.2 |
|  | Cefepime + Ciprofloxacin + Ethambutol | 8 | 0.2 |
|  | Ceftriaxone + Levofloxacin + Cycloserine | 8 | 0.2 |
|  | Meropenem + Cotrimoxazole + Metronidazole | 8 | 0.2 |
|  | Piperacillin + Clindamycin + Ethambutol | 8 | 0.2 |
|  | Cefepime + Levofloxacin + Rifampicin | 7 | 0.2 |
|  | Ceftazidime + Moxifloxacin + Metronidazole | 7 | 0.2 |
|  | Cefepime + Cotrimoxazole + Ethambutol | 6 | 0.1 |
|  | Ceftazidime + Erythromycin + Ethambutol | 6 | 0.1 |
|  | Ceftazidime + Erythromycin + Metronidazole | 6 | 0.1 |
|  | Ceftriaxone + Ciprofloxacin + Ethambutol | 6 | 0.1 |
|  | Ceftriaxone + Levofloxacin + Isoniazid | 6 | 0.1 |
|  | Ceftriaxone + Moxifloxacin + Rifampicin | 6 | 0.1 |
|  | Cefuroxime + Levofloxacin + Metronidazole | 6 | 0.1 |
|  | Flomoxef + Erythromycin + Metronidazole | 6 | 0.1 |
|  | Imipenem + Ciprofloxacin + Metronidazole | 6 | 0.1 |
|  | Imipenem + Levofloxacin + Metronidazole | 6 | 0.1 |
|  | Amoxicillin + Ciprofloxacin + Isoniazid | 5 | 0.1 |
|  | Ampicillin + Moxifloxacin + Metronidazole | 5 | 0.1 |
|  | Cefazolin + Levofloxacin + Isoniazid | 5 | 0.1 |
|  | Cefepime + Erythromycin + Ethambutol | 5 | 0.1 |
|  | Cefobactam + Cotrimoxazole + Metronidazole | 5 | 0.1 |
|  | Cefoxitin + Levofloxacin + Metronidazole | 5 | 0.1 |
|  | Ceftazidime + Levofloxacin + Ethambutol | 5 | 0.1 |
|  | Ceftizoxime + Cotrimoxazole + Metronidazole | 5 | 0.1 |
|  | Ceftriaxone + Erythromycin + Isoniazid | 5 | 0.1 |
|  | Ceftriaxone + Levofloxacin + Rifampicin | 5 | 0.1 |
|  | doripenem + Levofloxacin + Metronidazole | 5 | 0.1 |
|  | Ertapenem + Ciprofloxacin + Metronidazole | 5 | 0.1 |
|  | Meropenem + Moxifloxacin + Metronidazole | 5 | 0.1 |
|  | Amoxicillin + Clindamycin + Ethambutol | 4 | 0.1 |
|  | Amoxicillin + Moxifloxacin + Metronidazole | 4 | 0.1 |
|  | Ampicillin + Ciprofloxacin + Isoniazid | 4 | 0.1 |
|  | Cefazolin + Ciprofloxacin + Ethambutol | 4 | 0.1 |
|  | Cefazolin + Ciprofloxacin + Rifampicin | 4 | 0.1 |
|  | Cefobactam + Clindamycin + Ethambutol | 4 | 0.1 |
|  | Cefobactam + Erythromycin + Metronidazole | 4 | 0.1 |
|  | Cefoperazone + Clindamycin + Metronidazole | 4 | 0.1 |
|  | Cefotaxime + Clindamycin + Metronidazole | 4 | 0.1 |
|  | Cefotaxime + Erythromycin + Ethambutol | 4 | 0.1 |
|  | Cefotetan + Cotrimoxazole + Metronidazole | 4 | 0.1 |
|  | Ceftazidime + Ciprofloxacin + Isoniazid | 4 | 0.1 |
|  | Ceftizoxime + Levofloxacin + Cycloserine | 4 | 0.1 |
|  | Ceftriaxone + Azithromycin + Ethambutol | 4 | 0.1 |
|  | Ceftriaxone + Ciprofloxacin + Isoniazid | 4 | 0.1 |
|  | Ceftriaxone + Moxifloxacin + Ethambutol | 4 | 0.1 |
|  | Ceftriaxone + Moxifloxacin + Isoniazid | 4 | 0.1 |
|  | Ertapenem + Levofloxacin + Metronidazole | 4 | 0.1 |
|  | Flomoxef + Moxifloxacin + Metronidazole | 4 | 0.1 |
|  | Piperacillin + Ciprofloxacin + Ethambutol | 4 | 0.1 |
|  | Piperacillin + Ciprofloxacin + Rifampicin | 4 | 0.1 |
|  | Piperacillin + Cotrimoxazole + Ethambutol | 4 | 0.1 |
|  | Cefazolin + Erythromycin + Ethambutol | 3 | 0.1 |
|  | Cefazolin + Erythromycin + Metronidazole | 3 | 0.1 |
|  | Cefepime + Clindamycin + Ethambutol | 3 | 0.1 |
|  | Cefepime + Levofloxacin + Isoniazid | 3 | 0.1 |
|  | Cefepime + Moxifloxacin + Pyrazinamide | 3 | 0.1 |
|  | Cefobactam + Moxifloxacin + Ethambutol | 3 | 0.1 |
|  | Cefoperazone + Cotrimoxazole + Metronidazole | 3 | 0.1 |
|  | Cefoxitin + Erythromycin + Ethambutol | 3 | 0.1 |
|  | Ceftizoxime + Ciprofloxacin + Ethambutol | 3 | 0.1 |
|  | Ceftizoxime + Erythromycin + Rifampicin | 3 | 0.1 |
|  | Ceftizoxime + Levofloxacin + Ethambutol | 3 | 0.1 |
|  | Ceftriaxone + Ciprofloxacin + Rifampicin | 3 | 0.1 |
|  | Ceftriaxone + Clindamycin + Pyrazinamide | 3 | 0.1 |
|  | Ceftriaxone + Cotrimoxazole + Rifampicin | 3 | 0.1 |
|  | Ceftriaxone + Erythromycin + Rifampicin | 3 | 0.1 |
|  | Ceftriaxone + Moxifloxacin + Cycloserine | 3 | 0.1 |
|  | Cefuroxime + Ciprofloxacin + Ethambutol | 3 | 0.1 |
|  | Imipenem + Cotrimoxazole + Metronidazole | 3 | 0.1 |
|  | Imipenem + Erythromycin + Metronidazole | 3 | 0.1 |
|  | Meropenem + Levofloxacin + Ethambutol | 3 | 0.1 |
|  | Meropenem + Levofloxacin + Isoniazid | 3 | 0.1 |
|  | Piperacillin + Levofloxacin + Isoniazid | 3 | 0.1 |
|  | Piperacillin + Levofloxacin + Rifampicin | 3 | 0.1 |
|  | Piperacillin + Moxifloxacin + Cycloserine | 3 | 0.1 |
|  | Amoxicillin + Ciprofloxacin + Ethambutol | 2 | 0.0 |
|  | Amoxicillin + Clindamycin + Pyrazinamide | 2 | 0.0 |
|  | Amoxicillin + Cotrimoxazole + (soniazid | 2 | 0.0 |
|  | Amoxicillin + Levofloxacin + Cycloserine | 2 | 0.0 |
|  | Amoxicillin + Levofloxacin + Ethambutol | 2 | 0.0 |
|  | Amoxicillin + Moxifloxacin + Ethambutol | 2 | 0.0 |
|  | Ampicillin + Clindamycin + Metronidazole | 2 | 0.0 |
|  | ampicilin + Erythromycin + Ethambutol | 2 | 0.0 |
|  | Ampicillin + Levofloxacin + Isoniazid | 2 | 0.0 |
|  | Cefazolin + Cotrimoxazole + Ethambutol | 2 | 0.0 |
|  | Cefazolin + Levofloxacin + Ethambutol | 2 | 0.0 |
|  | Cefazolin + Levofloxacin + Rifampicin | 2 | 0.0 |
|  | Cefepime + Clindamycin + Rifampicin | 2 | 0.0 |
|  | Cefepime + Cotrimoxazole + Rifampicin | 2 | 0.0 |
|  | Cetepime + Erythromycin + Isoniazid | 2 | 0.0 |
|  | Cefepime + Moxifloxacin + Cycloserine | 2 | 0.0 |
|  | Cefepime + Moxifloxacin + Isoniazid | 2 | 0.0 |
|  | Cefobactam + Erythromycin + Ethambutol | 2 | 0.0 |
|  | Cefoperazone + Ciprofloxacin + Ethambutol | 2 | 0.0 |
|  | Cefoperazone + Erythromycin + Metronidazole | 2 | 0.0 |
|  | Cefotaxime + Azithromycin + Metronidazole | 2 | 0.0 |
|  | Cefotaxime + Clindamycin + Ethambutol | 2 | 0.0 |
|  | Cefotaxime + Cotrimoxazole + Metronidazole | 2 | 0.0 |
|  | Cefotaxime + Erythromycin + Metronidazole | 2 | 0.0 |
|  | Cefotetan + Clindamycin + Metronidazole | 2 | 0.0 |
|  | Cefotetan + Levofloxacin + Cycloserine | 2 | 0.0 |
|  | Cefotetan + Moxifloxacin + Metronidazole | 2 | 0.0 |
|  | Cefoxitin + Moxifloxacin + Metronidazole | 2 | 0.0 |
|  | Ceftazidime + Clindamycin + Ethambutol | 2 | 0.0 |
|  | Ceftazidime + Clindamycin + Metronidazole | 2 | 0.0 |
|  | Ceftriaxone + Erythromycin + Pyrazinamide | 2 | 0.0 |
|  | Ceftriaxone + Levofloxacin + Pyrazinamide | 2 | 0.0 |
|  | Ceftriaxone + Moxifloxacin + Pyrazinamide | 2 | 0.0 |
|  | Ceftriaxone + Tosufloxacin + Metronidazole | 2 | 0.0 |
|  | Cefuroxime + Cotrimoxazole + Metronidazole | 2 | 0.0 |
|  | Flomoxef + Ciprofloxacin + Isoniazid | 2 | 0.0 |
|  | Flomoxef + Levofloxacin + Isoniazid | 2 | 0.0 |
|  | Flomoxef + Moxifloxacin + Ethambutol | 2 | 0.0 |
|  | Imipenem + Cotrimoxazole + Isoniazid | 2 | 0.0 |
|  | Imipenem + Erythromycin + Ethambutol | 2 | 0.0 |
|  | Imipenem + Moxifloxacin + Ethambutol | 2 | 0.0 |
|  | Imipenem + Moxifloxacin + Metronidazole | 2 | 0.0 |
|  | Meropenem + Ciprofloxacin + Isoniazid | 2 | 0.0 |
|  | Meropenem + Cotrimoxazole + Ethambutol | 2 | 0.0 |
|  | Meropenem + Cotrimoxazole + Isoniazid | 2 | 0.0 |
|  | Meropenem + Cotrimoxazole + Rifampicin | 2 | 0.0 |
|  | Meropenem + Erythromycin + Ethambutol | 2 | 0.0 |
|  | Meropenem + Moxifloxacin + Rifampicin | 2 | 0.0 |
|  | Penicillin + Ciprofloxacin + Metronidazole | 2 | 0.0 |
|  | Piperacillin + Azithromycin + Metronidazole | 2 | 0.0 |
|  | Piperacillin + Cotrimoxazole + Rifampicin | 2 | 0.0 |
|  | Piperacillin + Erythromycin + Rifampicin | 2 | 0.0 |
|  | Piperacillin + Streptomycin + Metronidazole | 2 | 0.0 |
|  | Amoxicillin + Azithromycin + Ethambutol | 2 | 0.0 |
|  | Amoxicillin + Azithromycin + Metronidazole | 2 | 0.0 |
|  | Amoxicillin + Ciprofloxacin + Rifampicin | 1 | 0.0 |
|  | Amoxicillin + Clindamycin + Cycloserine | 1 | 0.0 |
|  | Amoxicillin + Clindamycin + Rifampicin | 1 | 0.0 |
|  | Amoxicillin + Erythromycin + Ethambutol | 1 | 0.0 |
|  | Amoxicillin + Erythromycin + Isoniazid | 1 | 0.0 |
|  | Amoxicillin + Erythromycin + Pyrazinamide | 1 | 0.0 |
|  | Amoxicillin + Erythromycin + Rifampicin | 1 | 0.0 |
|  | Amoxicillin + Levofloxacin + Isoniazid | 1 | 0.0 |
|  | Amoxicillin + Moxifloxacin + Rifampicin | 1 | 0.0 |
|  | Amoxicillin + Streptomycin + Cycloserine | 1 | 0.0 |
|  | Ampicillin + Azithromycin + Ethambutol | 1 | 0.0 |
|  | Ampicillin + Ciprofloxacin + Rifampicin | 1 | 0.0 |
|  | Ampicillin + Clindamycin + Rifampicin | 1 | 0.0 |
|  | Ampicillin + Levofloxacin + Ethambutol | 1 | 0.0 |
|  | Ampicillin + Moxifloxacin + Rifampicin | 1 | 0.0 |
|  | Cefazolin + Azithromycin + Ethambutol | 1 | 0.0 |
|  | Cefazolin + Azithromycin + Metronidazole | 1 | 0.0 |
|  | Cefazolin + Cotrimoxazole + Rifampicin | 1 | 0.0 |
|  | Cefazolin + Erythromycin + Rifampicin | 1 | 0.0 |
|  | Cefazolin + Levofloxacin + Cycloserine | 1 | 0.0 |
|  | Cefazolin + Moxifloxacin + Ethambutol | 1 | 0.0 |
|  | Cefazolin + Moxifloxacin + Isoniazid | 1 | 0.0 |
|  | Cefepime + Azithromycin + Cycloserine | 1 | 0.0 |
|  | Cefepime + Azithromycin + Ethambutol | 1 | 0.0 |
|  | Cefepime + Azithromycin + Metronidazole | 1 | 0.0 |
|  | Cefepime + Cotrimoxazole + Cycloserine | 1 | 0.0 |
|  | Cefepime + Erythromycin + Rifampicin | 1 | 0.0 |
|  | Cefepime + Moxifloxacin + Rifampicin | 1 | 0.0 |
|  | Cefobactam + Azithromycin + Metronidazole | 1 | 0.0 |
|  | Cefobactam + Ciprofloxacin + Ethambutol | 1 | 0.0 |
|  | Cefobactam + Erythromycin + Pyrazinamide | 1 | 0.0 |
|  | Cefobactam + Levofloxacin + Ethambutol | 1 | 0.0 |
|  | Cefobactam + Tosufloxacin + Metronidazole | 1 | 0.0 |
|  | Cefoperazone + Azithromycin + Ethambutol | 1 | 0.0 |
|  | Cefoperazone + Azithromycin + Metronidazole | 1 | 0.0 |
|  | Cefoperazone + Ciprofloxacin + Rifampicin | 1 | 0.0 |
|  | Cefoperazone + Clindamycin + Ethambutol | 1 | 0.0 |
|  | Cefoperazone + Clindamycin + Rifampicin | 1 | 0.0 |
|  | Cefoperazone + Cotrimoxazole + Rifampicin | 1 | 0.0 |
|  | Cefoperazone + Erythromycin + Ethambutol | 1 | 0.0 |
|  | Cefoperazone + Erythromycin + Isoniazid | 1 | 0.0 |
|  | Cefoperazone + Levofloxacin + Isoniazid | 1 | 0.0 |
|  | Cefoperazone + Moxifloxacin + Metronidazole | 1 | 0.0 |
|  | Cefotaxime + Ciprofloxacin + Ethambutol | 1 | 0.0 |
|  | Cefotaxime + Ciprofloxacin + Isoniazid | 1 | 0.0 |
|  | Cefotaxime + Levofloxacin + Ethambutol | 1 | 0.0 |
|  | Cefotaxime + Moxifloxacin + Metronidazole | 1 | 0.0 |
|  | Cefotetan + Ciprofloxacin + Ethambutol | 1 | 0.0 |
|  | Cefotetan + Erythromycin + Metronidazole | 1 | 0.0 |
|  | Cefotetan + Levofloxacin + Ethambutol | 1 | 0.0 |
|  | Cefoxitin + Levofloxacin + Cycloserine | 1 | 0.0 |
|  | Ceftazidime + Ciprofloxacin + Ethambutol | 1 | 0.0 |
|  | Ceftazidime + Ciprofloxacin + Rifampicin | 1 | 0.0 |
|  | Ceftazidime + Cotrimoxazole + Rifampicin | 1 | 0.0 |
|  | Ceftazidime + Levofloxacin + Isoniazid | 1 | 0.0 |
|  | Ceftazidime + Levofloxacin + Rifampicin | 1 | 0.0 |
|  | Ceftazidime + Moxifloxacin + Cycloserine | 1 | 0.0 |
|  | Ceftazidime + Moxifloxacin + Pyrazinamide | 1 | 0.0 |
|  | Ceftizoxime + Erythromycin + Pyrazinamide | 1 | 0.0 |
|  | Ceftizoxime + Moxifloxacin + Metronidazole | 1 | 0.0 |
|  | Ceftriaxone + Clindamycin + Isoniazid | 1 | 0.0 |
|  | Ceftriaxone + Clindamycin + Rifampicin | 1 | 0.0 |
|  | Ceftriaxone + Erythromycin + Cycloserine | 1 | 0.0 |
|  | Cefuroxime + Ciprofloxacin + Rifampicin | 1 | 0.0 |
|  | Cefuroxime + Clindamycin + Metronidazole | 1 | 0.0 |
|  | Cefuroxime + Levofloxacin + Ethambutol | 1 | 0.0 |
|  | doripenem + Ciprofloxacin + Metronidazole | 1 | 0.0 |
|  | doripenem + Clindamycin + Metronidazole | 1 | 0.0 |
|  | doripenem + Cotrimoxazole + Rifampicin | 1 | 0.0 |
|  | doripenem + Erythromycin + Metronidazole | 1 | 0.0 |
|  | doripenem + Moxifloxacin + Metronidazole | 1 | 0.0 |
|  | Ertapenem + Moxifloxacin + Metronidazole | 1 | 0.0 |
|  | Flomoxef + Azithromycin + Ethambutol | 1 | 0.0 |
|  | Flomoxef + Clindamycin + Metronidazole | 1 | 0.0 |
|  | Flomoxef + Cotrimoxazole + Rifampicin | 1 | 0.0 |
|  | Flomoxef + Erythromycin + Pyrazinamide | 1 | 0.0 |
|  | Flomoxef + Levofloxacin + Cycloserine | 1 | 0.0 |
|  | Flomoxef + Levofloxacin + Ethambutol | 1 | 0.0 |
|  | Flomoxef + Levofloxacin + Pyrazinamide | 1 | 0.0 |
|  | Flomoxef + Moxifloxacin + Pyrazinamide | 1 | 0.0 |
|  | Imipenem + Azithromycin + Metronidazole | 1 | 0.0 |
|  | Imipenem + Ciprofloxacin + Ethambutol | 1 | 0.0 |
|  | Imipenem + Levofloxacin + Ethambutol | 1 | 0.0 |
|  | Meropenem + Azithromycin + Metronidazole | 1 | 0.0 |
|  | Meropenem + Ciprofloxacin + Ethambutol | 1 | 0.0 |
|  | Meropenem + Erythromycin + Metronidazole | 1 | 0.0 |
|  | Meropenem + Levofloxacin + Rifampicin | 1 | 0.0 |
|  | Meropenem + Moxifloxacin + Ethambutol | 1 | 0.0 |
|  | Meropenem + Moxifloxacin + Isoniazid | 1 | 0.0 |
|  | Meropenem + Streptomycin + Ethambutol | 1 | 0.0 |
|  | Nafcillin + Ciprofloxacin + Metronidazole | 1 | 0.0 |
|  | Penicillin + Clindamycin + Metronidazole | 1 | 0.0 |
|  | Piperacillin + Azithromycin + Ethambutol | 1 | 0.0 |
|  | Piperacillin + Erythromycin + Isoniazid | 1 | 0.0 |
|  | Piperacillin + Levofloxacin + Pyrazinamide | 1 | 0.0 |
|  | Piperacillin + Moxifloxacin + Isoniazid | 1 | 0.0 |
|  | Cefuroxime + Ciprofloxacin + Riffampicin | 1 | 0.0 |
|  | Cefuroxime + Clindamycin + Metronidazole | 1 | 0.0 |
|  | Cefuroxime + Levofloxacin + Ethambutol | 1 | 0.0 |
|  | doripenem + Ciprofloxacin + Metronidazole | 1 | 0.0 |
|  | doripenem + Clindamycin + Metronidazole | 1 | 0.0 |
|  | doripenem + Cotrimoxazole + Rifampicin | 1 | 0.0 |
|  | doripenem + Erythromycin + Metronidazole | 1 | 0.0 |
|  | doripenem + Moxifloxacin + Metronidazole | 1 | 0.0 |
|  | Ertapenem + Moxifloxacin + Metronidazole | 1 | 0.0 |
|  | Flomoxef + Azithromycin + Ethambutol | 1 | 0.0 |
|  | Flomoxef + Clindamycin + Metronidazole | 1 | 0.0 |
|  | Flomoxef + Cotrimoxazole + Rifampicin | 1 | 0.0 |
|  | Flomoxef + Erythromycin + Pyrazinamide | 1 | 0.0 |
|  | Flomoxef + Levofloxacin + Cycloserine | 1 | 0.0 |
|  | Flomoxef + Levofloxacin + Ethambutol | 1 | 0.0 |
|  | Flomoxef + Levofloxacin + Pyrazinamide | 1 | 0.0 |
|  | Flomoxef + Moxifloxacin + Pyrazinamide | 1 | 0.0 |
|  | Imipenem + Azithromycin + Metronidazole | 1 | 0.0 |
|  | Imipenem + Ciprofloxacin + Ethambutol | 1 | 0.0 |
|  | Imipenem + Levofloxacin + Ethambutol | 1 | 0.0 |
|  | Meropenem + Azithromycin + Metronidazole | 1 | 0.0 |
|  | Meropenem + Ciprofloxacin + Ethambutol | 1 | 0.0 |
|  | Meropenem + Erythromycin + Metronidazole | 1 | 0.0 |
|  | Meropenem + Levofloxacin + Rifampicin | 1 | 0.0 |
|  | Meropenem + Moxifloxacin + Ethambutol | 1 | 0.0 |
|  | Meropener + Moxifloxacin + Isoniazid | 1 | 0.0 |
|  | Meropenem + Streptomycin + Ethambutol | 1 | 0.0 |
|  | Nafcillin + Ciprofloxacin + Metronidazole | 1 | 0.0 |
|  | Penicillin + Clindamycin + Metronidazole | 1 | 0.0 |
|  | Piperacillin + Azithromycin + Ethambutol | 1 | 0.0 |
|  | Piperacillin + Erythromycin + Isoniazid | 1 | 0.0 |
|  | Piperacillin + Levofloxacin + Pyrazinamide | 1 | 0.0 |
|  | Piperacillin + Moxifloxacin + Isoniazid | 1 | 0.0 |
|  | Piperacillin + Moxifloxacin + Pyrazinamide | 1 | 0.0 |
|  | Piperacillin + Moxifloxacin + Rifampicin | 1 | 0.0 |
|  | Piperacillin + Streptomycin + Ethambutol | 1 | 0.0 |
|  | Total | 4455 | 100 |
